# METhodological RadiomICs Score (METRICS): a quality scoring tool for radiomics research endorsed by EuSoMII

**DOI:** 10.1186/s13244-023-01572-w

**Published:** 2024-01-17

**Authors:** Burak Kocak, Tugba Akinci D’Antonoli, Nathaniel Mercaldo, Angel Alberich-Bayarri, Bettina Baessler, Ilaria Ambrosini, Anna E. Andreychenko, Spyridon Bakas, Regina G. H. Beets-Tan, Keno Bressem, Irene Buvat, Roberto Cannella, Luca Alessandro Cappellini, Armando Ugo Cavallo, Leonid L. Chepelev, Linda Chi Hang Chu, Aydin Demircioglu, Nandita M. deSouza, Matthias Dietzel, Salvatore Claudio Fanni, Andrey Fedorov, Laure S. Fournier, Valentina Giannini, Rossano Girometti, Kevin B. W. Groot Lipman, Georgios Kalarakis, Brendan S. Kelly, Michail E. Klontzas, Dow-Mu Koh, Elmar Kotter, Ho Yun Lee, Mario Maas, Luis Marti-Bonmati, Henning Müller, Nancy Obuchowski, Fanny Orlhac, Nikolaos Papanikolaou, Ekaterina Petrash, Elisabeth Pfaehler, Daniel Pinto dos Santos, Andrea Ponsiglione, Sebastià Sabater, Francesco Sardanelli, Philipp Seeböck, Nanna M. Sijtsema, Arnaldo Stanzione, Alberto Traverso, Lorenzo Ugga, Martin Vallières, Lisanne V. van Dijk, Joost J. M. van Griethuysen, Robbert W. van Hamersvelt, Peter van Ooijen, Federica Vernuccio, Alan Wang, Stuart Williams, Jan Witowski, Zhongyi Zhang, Alex Zwanenburg, Renato Cuocolo

**Affiliations:** 1 Department of Radiology, University of Health Sciences, Basaksehir Cam and Sakura City Hospital, Basaksehir, Istanbul, Turkey; 2 Institute of Radiology and Nuclear Medicine, Cantonal Hospital Baselland, Liestal, Switzerland; 3 Department of Radiology, Massachusetts General Hospital, Boston, MA USA; 4 Quantitative Imaging Biomarkers in Medicine (Quibim), Valencia, Spain; 5 Department of Diagnostic and Interventional Radiology, University Hospital Würzburg, Würzburg, Germany; 6 Department of Translational Research, Academic Radiology, University of Pisa, Pisa, Italy; 7 Laboratory for Digital Public Health Technologies, ITMO University, St. Petersburg, Russian Federation; 8 Division of Computational Pathology, Department of Pathology and Laboratory Medicine, School of Medicine, Indiana University, Indianapolis, IN USA; 9 Center for Federated Learning in Precision Medicine, Indiana University, Indianapolis, IN USA; 10 Department of Radiology, The Netherlands Cancer Institute, Amsterdam, the Netherlands; 11 GROW School for Oncology and Developmental Biology, Maastricht University Medical Center, Maastricht, the Netherlands; 12 Institute of Regional Health Research, University of Southern Denmark, Odense, Denmark; 13 Department of Radiology, Charité – Universitätsmedizin Berlin, Corporate Member of Freie Universität Berlin and Humboldt- Universität zu Berlin, Berlin, Germany; 14 Berlin Institute of Health at Charité - Universitätsmedizin Berlin, Berlin, Germany; 15 Institut Curie, Inserm, PSL University, Laboratory of Translational Imaging in Oncology, Orsay, France; 16 Section of Radiology - Department of Biomedicine, Neuroscience and Advanced Diagnostics (BiND), University of Palermo, Palermo, Italy; 17 Department of Biomedical Sciences, Humanitas University, Pieve Emanuele, Milan, Italy; 18 Division of Radiology, Istituto Dermopatico dell’Immacolata (IDI) IRCCS, Rome, Italy; 19 Joint Department of Medical Imaging, University Health Network, University of Toronto, Toronto, Canada; 20 The Russell H. Morgan Department of Radiology and Radiological Science, Johns Hopkins University School of Medicine, Baltimore, USA; 21 Institute of Diagnostic and Interventional Radiology and Neuroradiology, University Hospital , Essen, Germany; 22 Division of Radiotherapy and Imaging, The Institute of Cancer Research, London, UK; 23 Department of Imaging, The Royal Marsden National Health Service (NHS) Foundation Trust, London, UK; 24 Department of Radiology, University Hospital Erlangen, Erlangen, Germany; 25 Department of Radiology, Brigham and Women’s Hospital, Harvard Medical School, Boston, MA USA; 26 Department of Radiology, Université Paris Cité, AP-HP, Hôpital Européen Georges Pompidou, PARCC UMRS 970, INSERM, Paris, France; 27 Department of Surgical Sciences, University of Turin, Turin, Italy; 28 Institute of Radiology, Department of Medicine, University of Udine, University Hospital S. Maria della Misericordia, Udine, Italy; 29 Department of Thoracic Oncology, Netherlands Cancer Institute, Amsterdam, the Netherlands; 30 Department of Neuroradiology, Karolinska University Hospital, Stockholm, Sweden; 31 Department of Clinical Science, Division of Radiology, Intervention and Technology (CLINTEC), Karolinska Institutet, Stockholm, Sweden; 32 Department of Radiology, Medical School, University of Crete, Heraklion, Greece; 33 Department of Radiology, St Vincent’s University Hospital, Dublin, Ireland; 34 Insight Centre for Data Analytics, UCD, Dublin, Ireland; 35 School of Medicine, University College Dublin, Dublin, Ireland; 36 Department of Medical Imaging, University Hospital of Heraklion, Crete, Greece; 37 Department of Radiology, School of Medicine, University of Crete, Heraklion, Crete, Greece; 38 Computational Biomedicine Laboratory, Institute of Computer Science, FORTH, Heraklion, Crete, Greece; 39 Department of Radiology, Royal Marsden Hospital, Sutton, UK; 40 Department of Diagnostic and Interventional Radiology, Faculty of Medicine and Medical Center-University of Freiburg, Freiburg, Germany; 41 Department of Radiology and Center for Imaging Science, Samsung Medical Center, Sungkyunkwan University School of Medicine, Seoul, South Korea; 42 Department of Health Sciences and Technology, Samsung Advanced Institute for Health Science & Technology (SAIHST), Sungkyunkwan University, Seoul, South Korea; 43 Department of Radiology & Nuclear Medicine, Amsterdam UMC Location University of Amsterdam, Meibergdreef 9, Amsterdam, the Netherlands; 44 Medical Imaging Department and Biomedical Imaging Research Group, Hospital Universitario y Politécnico La Fe and Health Research Institute, Valencia, Spain; 45 University of Applied Sciences of Western Switzerland (HES-SO Valais), Sierra, Switzerland; 46 Department of Radiology and Medical Informatics, University of Geneva (UniGe), Geneva, Switzerland; 47 Quantitative Health Sciences, Lerner Research Institute, Cleveland Clinic, Cleveland, OH USA; 48 Computational Clinical Imaging Group, Centre for the Unknown, Champalimaud Foundation, Lisbon, Portugal; 49 Department of Radiology, Royal Marsden Hospital and The Institute of Cancer Research, London, UK; 50 Radiology department, Research Institute of Pediatric Oncology and Hematology n. a. L.A. Durnov, National Medical Research Center of Oncology n. a. N.N. Blokhin Ministry of Health of Russian Federation, Moscow, Russia; 51 Medical Department IRA-Labs, Moscow, Russia; 52 Institute for advanced simulation (IAS-8): Machine learning and data analytics, Forschungszentrum Jülich, Jülich, Germany; 53 Department of Radiology, University Hospital of Cologne, Cologne, Germany; 54 Institute for Diagnostic and Interventional Radiology, Goethe-University Frankfurt Am Main, Frankfurt, Germany; 55 Department of Advanced Biomedical Sciences, University of Naples Federico II, Naples, Italy; 56 Department of Radiation Oncology, Complejo Hospitalario Universitario de Albacete, Albacete, Spain; 57 Department of Biomedical Sciences for Health, Università degli Studi di Milano, Milan, Italy; 58 Unit of Radiology, IRCCS Policlinico San Donato, San Donato Milanese, Milan, Italy; 59 Computational Imaging Research Lab, Department of Biomedical Imaging and Image-guided Therapy, Medical University of Vienna, Vienna, Austria; 60 Department of Radiation Oncology, University Medical Center Groningen, University of Groningen, Groningen, the Netherlands; 61 Department of Radiotherapy, Maastro Clinic, Maastricht, the Netherlands; 62 School of Medicine, Vita-Salute San Raffaele University, Milan, Italy; 63 Department of Computer Science, Université de Sherbrooke, Sherbrooke, Canada; 64 Centre de recherche du Centre hospitalier universitaire de Sherbrooke, Sherbrooke, Canada; 65 Department of Radiation Oncology, University Medical Center Groningen, University of Groningen, Groningen, the Netherlands; 66 Department of Radiology, University Medical Center Utrecht, Utrecht University, Utrecht, the Netherlands; 67 Department of Radiotherapy, University of Groningen, University Medical Center Groningen, Groningen, the Netherlands; 68 Section of Radiology, Department of Biomedicine, Neuroscience and Advanced Diagnosis (Bi.N.D), University of Palermo, Palermo, 90127 Italy; 69 Centre for Medical Imaging & Centre for Brain Research, Faculty of Medical and Health Sciences, Auckland Bioengineering Institute, University of Auckland, Auckland, New Zealand; 70 Department of Radiology, Norfolk & Norwich University Hospital, Colney Lane, Norwich, Norfolk, UK; 71 Department of Radiology, New York University Grossman School of Medicine, New York, USA; 72 School of Information and Communication Technology, Griffith University, Nathan, Brisbane, Australia; 73 National Center for Tumor Diseases (NCT/UCC), Dresden, Germany; 74 OncoRay – National Center for Radiation Research in Oncology, Faculty of Medicine and University Hospital Carl Gustav Carus, Technische Universität Dresden, Helmholtz-Zentrum Dresden - Rossendorf, Dresden, Germany; 75 German Cancer Research Center (DKFZ), Heidelberg, Germany; 76 Department of Medicine, Surgery and Dentistry, University of Salerno, Baronissi, Italy

**Keywords:** Radiomics, Deep learning, Artificial intelligence, Machine learning, Guideline

## Abstract

**Purpose:**

To propose a new quality scoring tool, METhodological RadiomICs Score (METRICS), to assess and improve research quality of radiomics studies.

**Methods:**

We conducted an online modified Delphi study with a group of international experts. It was performed in three consecutive stages: Stage#1, item preparation; Stage#2, panel discussion among EuSoMII Auditing Group members to identify the items to be voted; and Stage#3, four rounds of the modified Delphi exercise by panelists to determine the items eligible for the METRICS and their weights. The consensus threshold was 75%. Based on the median ranks derived from expert panel opinion and their rank-sum based conversion to importance scores, the category and item weights were calculated.

**Result:**

In total, 59 panelists from 19 countries participated in selection and ranking of the items and categories. Final METRICS tool included 30 items within 9 categories. According to their weights, the categories were in descending order of importance: study design, imaging data, image processing and feature extraction, metrics and comparison, testing, feature processing, preparation for modeling, segmentation, and open science. A web application and a repository were developed to streamline the calculation of the METRICS score and to collect feedback from the radiomics community.

**Conclusion:**

In this work, we developed a scoring tool for assessing the methodological quality of the radiomics research, with a large international panel and a modified Delphi protocol. With its conditional format to cover methodological variations, it provides a well-constructed framework for the key methodological concepts to assess the quality of radiomic research papers.

**Critical relevance statement:**

A quality assessment tool, METhodological RadiomICs Score (METRICS), is made available by a large group of international domain experts, with transparent methodology, aiming at evaluating and improving research quality in radiomics and machine learning.

**Key points:**

• A methodological scoring tool, METRICS, was developed for assessing the quality of radiomics research, with a large international expert panel and a modified Delphi protocol.

• The proposed scoring tool presents expert opinion-based importance weights of categories and items with a transparent methodology for the first time.

• METRICS accounts for varying use cases, from handcrafted radiomics to entirely deep learning-based pipelines.

• A web application has been developed to help with the calculation of the METRICS score (https://metricsscore.github.io/metrics/METRICS.html) and a repository created to collect feedback from the radiomics community (https://github.com/metricsscore/metrics).

**Graphical Abstract:**

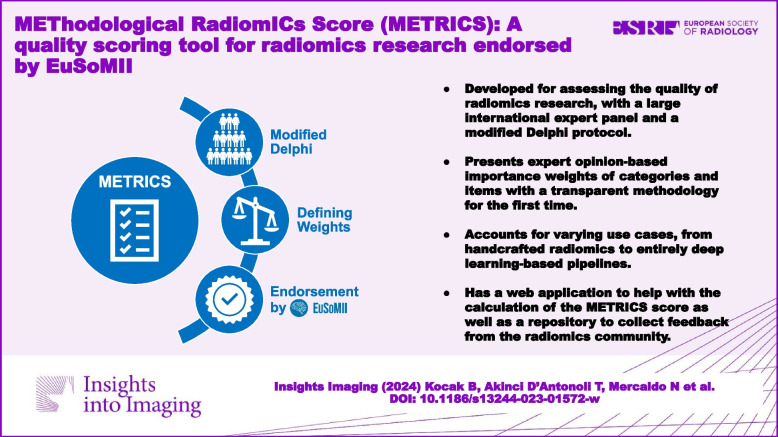

**Supplementary Information:**

The online version contains supplementary material available at 10.1186/s13244-023-01572-w.

## Introduction

Radiomics is an evolving field of image analysis technique for extracting quantitative features from medical images with the premise of building predictive models and assisting clinical decision-making [1]. Since its introduction into medicine more than a decade ago, an exponential number of radiomics-related articles have been published yearly [2]. However, a growing translational gap exists between radiomics research and clinical practice [3, 4]. One of the main reasons for this issue is the poor quality of research methodology, including but not limited to, poor study design, inadequate description of image segmentation, feature extraction or model building methodology, lack of generalizability, lack of data, and model and code sharing practices, all of which ultimately limit the reproducibility of the proposed radiomics models [3, 5–9].

In 2017, Lambin et al. [10] proposed the radiomics quality score (RQS), a set of assessment criteria covering the radiomics workflow to improve the quality of radiomics research. Since then, many systematic reviews have been published applying the RQS to published research to examine the quality of radiomics studies [11]. Nevertheless, some RQS item definitions may lead to ambiguity and the applicability of the items can be limited based on different characteristics of the study design, which may negatively affect the reproducibility of the score even among experts in the field [11–13]. In addition, as shown previously [12], a high RQS score does not always guarantee high quality of a study or lack of significant bias [14]. Furthermore, this assessment system was developed by a small group of researchers and the development process was not detailed in-depth in terms of how it deals with the relative importance of each item that contributes to overall radiomics research quality.

Recently, the CheckList for EvaluAtion of Radiomics Research (CLEAR) guideline for reporting radiomics studies that covers the entire life cycle of optimal radiomics research was published and endorsed by the European Society of Radiology (ESR) and European Society of Medical Imaging Informatics (EuSoMII) [15]. The CLEAR reporting guideline has great potential to improve the quality of reporting in radiomics papers, which would ultimately lead to an improvement in research quality. Nevertheless, reporting guidelines are not assessment tools or instruments for measuring research quality [16, 17]. Thus, the need remains for an easy-to-use, reproducible assessment system for radiomics research. In this paper, we propose a new quality assessment tool, METhodological RadiomICs Score (METRICS), which was developed by a large group of international experts in the field and is easy to use, specifically aimed at improving methodological quality of radiomics research.

## Material and methods

### Design and development

As there is no guidance for developing scoring systems, the recommendations for developing reporting guidelines were followed [18]*.* Therefore, a steering committee (T.A.D., B.K., and R.C.) was established first to organize and coordinate the development of METRICS.

To develop the METRICS tool, an online modified Delphi study with a group of international experts was planned. The process was organized in three stages. The steering committee members conducted the first stage (Stage#1), consisting of item preparation. The second stage (Stage#2) was held with the participation of a group of panelists from the EuSoMII Radiomics Auditing Group for discussion of the items to be voted on. The third stage (Stage#3) was carried out in four rounds by two separate groups of panelists to determine the METRICS items and their weights. The first three rounds of Stage#3 were aimed at determining which methodological items were eligible for METRICS. The items’ weights were then determined in the final round of Stage#3. Following each round, the panelists received structured feedback on the preceding round to reconcile individual opinions.

The surveys were open for at least 2 weeks in each round in Stage#3, and a reminder e-mail was sent 1 week, 3 days, and 1 day before the deadline. When necessary (e.g., when overlapping with major conferences or holidays), deadlines were extended to ensure a reasonable number of panelists was achieved.

The modified Delphi surveys were carried out using a computer-assisted web interviewing (CAWI) system, i.e., Google Forms (Google LLC). For online group discussions, online platforms, i.e., Google Docs (Google LLC) or WhatsApp (Meta Platforms Inc.), were used.

To simplify the calculation of the METRICS score, the development of an online calculation tool was planned. A GitHub repository was also planned for providing updates and gathering community feedback.

### Anonymity

Although the panelists voted independently, the voting rounds of the modified Delphi exercise were not anonymous to track panelists’ participation. Only the organizers had access to the panelists’ data, and they preserved the anonymity of the votes and their respective comments during and after the voting tasks (i.e., when feedback was provided after rounds).

### Informed consent

At the start of the Delphi questions, participants' informed consent was requested using the same form. Participants may have opted out of the study at any time. Those who indicated a desire to decline the survey were to be deleted from future invitations. Only while the round was active, panelists could withdraw their votes.

### Consensus criteria

The vote for “strongly agree” and “agree” accounted for agreement and “strongly disagree” and “disagree” accounted for disagreement. The “neutral” votes were not included in either decision. The consensus was defined *a priori* as either agreement (agreement ≥75%) or disagreement (disagreement ≥75%) [19]. If there was no agreement or disagreement, it was referred to as "no consensus," and they were voted again. If “no consensus” items did not achieve agreement in the next voting, they were removed from the tool. The consensus items with disagreement were removed from the tool without further discussion.

### Recruitment of participants

Individuals having significant experience in radiomics, machine learning, deep learning, informatics, or related editorial tasks from various countries were invited via an e-mail describing the development plan of the METRICS tool and explaining its purpose. Members of the EuSoMII Radiomics Auditing Group (Group#1 panelists) were assigned to discussion panels in Stage#2 and Round#3 of Stage#3. Other invitees (Group#2 panelists) were assigned to modified Delphi voting rounds (i.e., Round#1, Round#2, and Round#4 of Stage#3).

### Modified Delphi

#### Stage#1 (preparation)

To identify potential items, a thorough and systematic literature review was conducted. Two members of the steering committee performed an independent literature search in PubMed using the following syntax to find the relevant checklists, guidelines, or tools: (radiomics) AND ((checklist) OR (guideline)). The search date was January 24, 2023. All entries and related publications, if accessible by the readers, were assessed to determine the currently available tools. All eligible documents found were independently evaluated by the entire steering committee to develop the initial template of METRICS.

Participants were requested to consider the following principles: *i*, there should be no overlap between items; *ii*, an ideal study should be able to achieve a perfect score (i.e., all points available or 100%), meaning that items should not be mutually exclusive; *iii*, items must be objectively defined, to increase reproducibility; *iv*, not only hand-crafted but also studies based on deep learning should be considered and item conditionality should be assessed accordingly; *v*, since this is a methodological scoring system, the items should be mainly related to the “Material and methods” and “Results” sections of a research paper; *vi*, while items should also aim at improving the methodological reproducibility and transparency of the studies, METRICS is not a reporting checklist; and *vii*, items should point out potential bias sources and help users to avoid them.

Considering the principles defined above, an initial draft was created with three organizers of the METRICS project. For any disagreement among the organizers, the decisions were made based on a majority vote.

#### Stage#2 (discussion with Group#1 panelists)

The items prepared by the organizers were presented to the EuSoMII Radiomics Auditing Group with the same principles and discussed online. This stage was an open discussion and not anonymous. The panelists were free to suggest adding, removing, merging, and modifying items.

#### Stage#3 (modified Delphi rounds)

##### Round#1 (item selection)

On a 5-point Likert scale (strongly agree; agree; neutral; disagree; strongly disagree), the Group#2 panelists were asked to rate the extent to which they agreed with the inclusion of each item on the METRICS tool. With a text box, participants were further asked for suggestions on the item's name and definition. In addition, a text box was provided at the end of each section for participants to suggest additional items. After this round, the Group#2 panelists were provided with a statistical summary of each item from Round#1, along with anonymous comments.

##### Round#2 (continued for item selection)

The same panelists as in Round#1 were invited to participate in Round#2. Panelists who were invited but did not respond to Round#1 were also invited to participate in Round#2. Using the same structure as Round#1, panelists were also presented with items that reached no consensus as well as new item or items suggested in previous round. They were asked to use the same 5-point Likert scale to express their level of agreement with the inclusion of each item in the METRICS tool. No new item proposal was asked in this round. After Round#2, the same panelists were provided with a statistical summary of each item from Round#2, along with anonymized comments.

##### Round#3 (group discussion with EuSoMII Radiomics Auditing Group)

The purpose of Round#3 was to discuss the results of the previous rounds, modify if necessary, and finalize the items to be included in the METRICS tool. It was held on online platforms (Google Docs and WhatsApp Group). All Group#1 panelists were invited. The discussion included both agreed and unresolved topics. Any modification proposals were discussed and items were edited in consensus by the steering committee.

##### Round#4 (ranking of finalized items to determine the weights)

Group#2 panelists who participated in at least one of the first two rounds (Round#1 and Round#2) were invited to this round. The panelists were asked to rank the categories and then all items within each category in order of their importance in radiomics research. After Round#4, the same panelists were provided with an anonymized statistical summary of each item and category.

##### Pilot testing

We invited Group#1 panelists to test the usability and understandability of the online checklist. Also, the final METRICS tool was tested on studies from the literature, including a sample of different pipeline designs and aims (i.e., handcrafted radiomics, deep radiomics, and end-to-end deep learning; lesion characterization and region of interest segmentation).

### Statistical analysis

Descriptive statistics (i.e., median, interquartile range, percentage) were used to present the results. The ranks derived from hierarchical (i.e., multi-tiered) ranking with expert panel opinion were aggregated using their median value. Using the rank-sum method [20, 21], median ranks were first converted to importance scores with the following formula: *Score = (N+1) - Rank*, where *N* is the total number of categories or total number of items within a category. The category weights were then rescaled to 1. The final weights of each item were computed as the product of the category and item weights (e.g., [weight of Category A] x [weight of Item#1 in Category A]). The items within the respective category went through the same rescaling procedure. The final METRICS score was calculated on a percentage scale, accounting for the conditionality of items and categories.

## Results

**Fig. 1:**
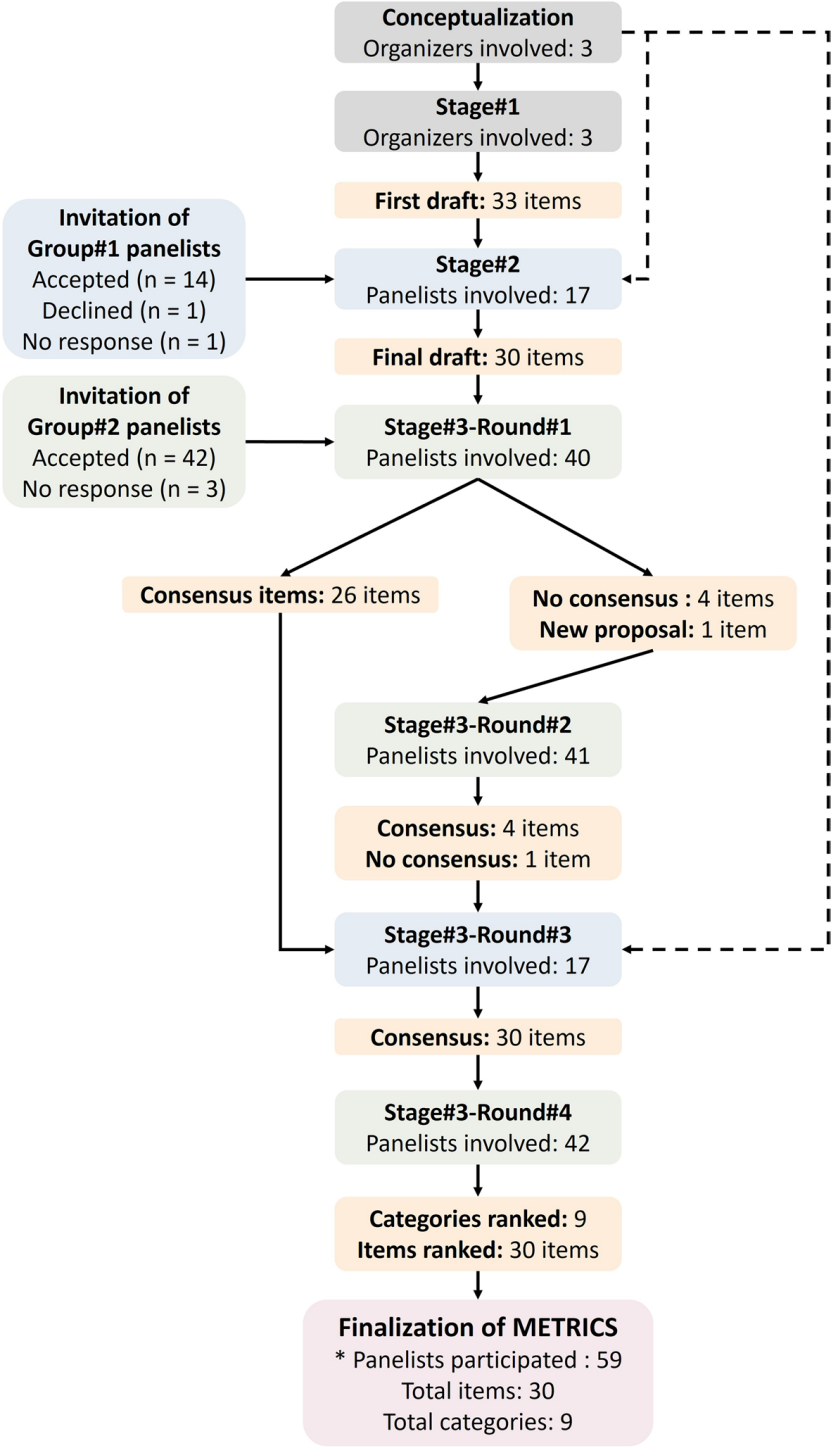
Key steps in the development of METRICS. Boxes related to stages and rounds are color-coded based on the main group of panelists involved. Dotted lines indicate the participation of organizers in the discussions in the relevant rounds as panelists. *Including organizers (i.e., steering committee members)

All key study steps are summarized with a flowchart in Fig. 1.

### Modified Delphi

**Fig. 2:**
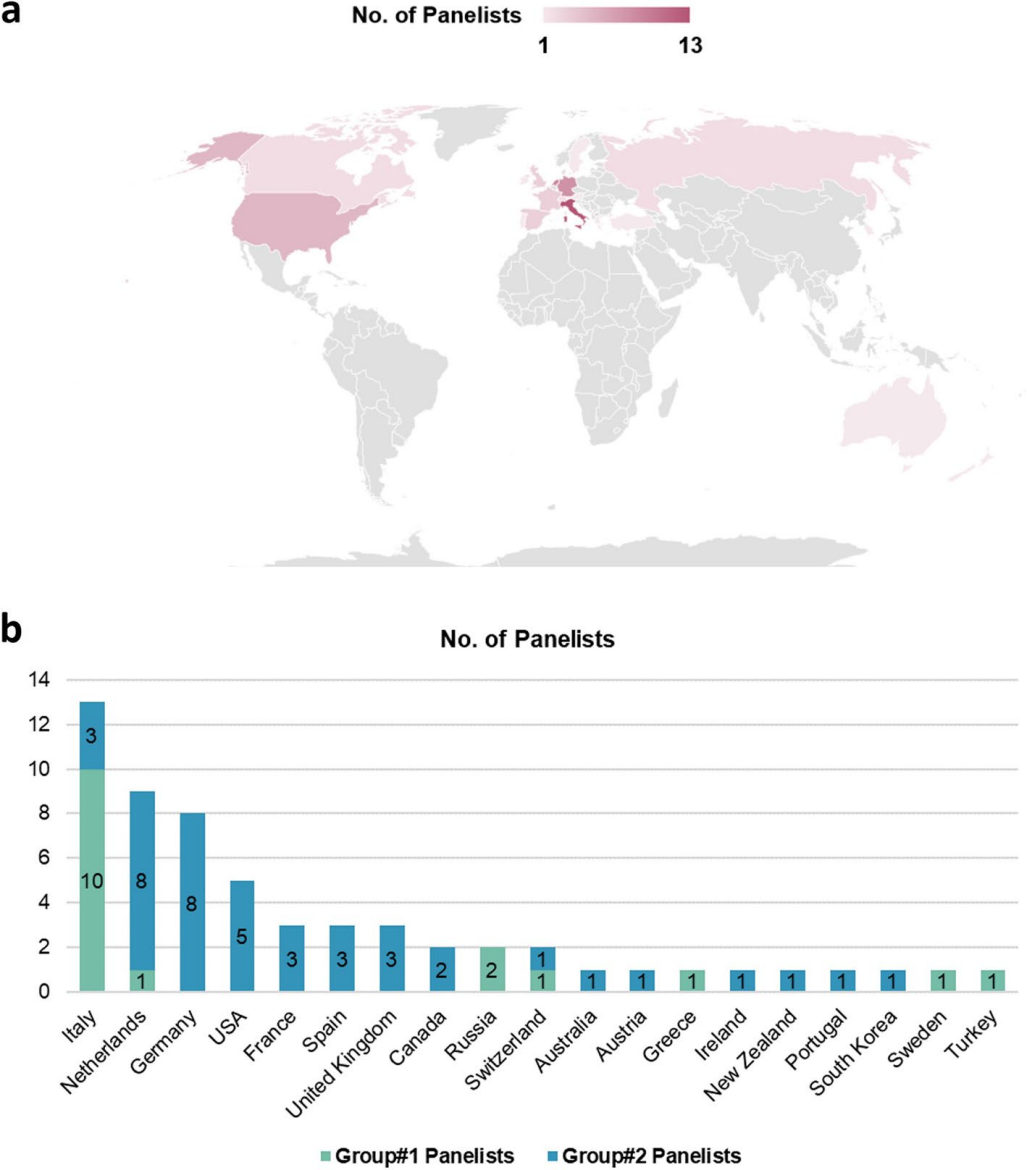
Country of panelists. **a** World map for distribution of 59 panelists including three organizers by country. **b** Countries by groups. Group#1, EuSoMII auditing group including three organizers participated in discussions at Stage#2 and Round#3 of Stage#3; Group#2, voters participated in Round#1, Round#2, and Round#4 of Stage#3. In case of multiple countries, the country of the first affiliation was considered

In total, the 3 steering committee members invited 61 experts to participate in this study, 56 of which accepted the invitation. In detail, 14 experts from the EuSoMII Radiomics Auditing Group (Group#1) accepted the invitation to participate in panel discussions (i.e., discussions at Stage#2 and Round#3 of Stage#3), together with the steering committee members. Furthermore, 42 experts (Group#2) accepted the invitation to perform Delphi voting (i.e., rating in Round#1 and Round#2; ranking in Round#4 of Stage#3). Country data of all participants is presented in Fig. 2.

The literature search resulted in 58 publications. After independent evaluation of the content of these publications by steering committee members, 16 relevant checklists, guidelines, or quality scoring tools were identified as potentially useful for designing a new quality scoring tool [7, 10, 22–35]. Based on the results of this literature review and previous experience, 33 items were initially drafted. These items were then reduced to 30 after discussion with the Group#1 panelists in Stage#2, as three were considered unclear or partly overlapping with other entries, with which they were merged.

**Fig. 3:**
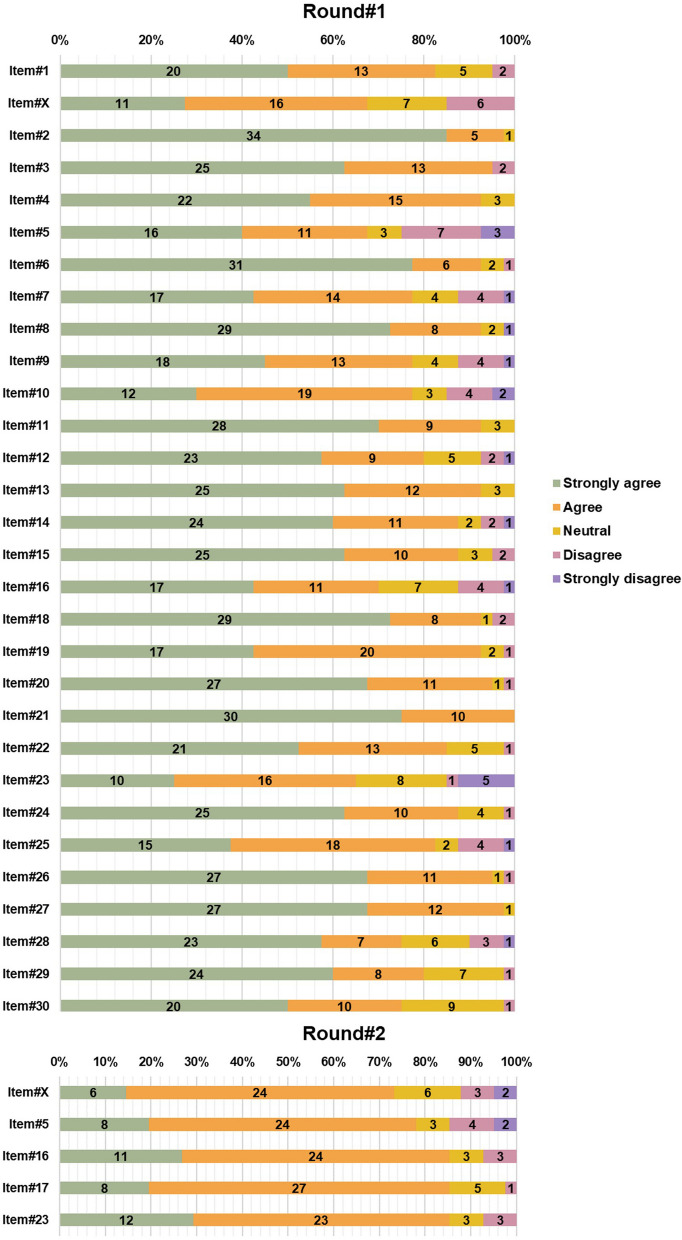
Rates from modified Delphi Round#1 and Round#2 of Stage#3. The number of the items matches those of the final METRICS tool. Item#X, i.e., prospective data collection, stands for the excluded item from the final METRICS tool. Please note Item#17 is missing in Round#1, which is the proposed item in Round#1 to be voted in Round#2

The 30 items obtained after Stage#2 discussion were presented to the Group#2 panelists for the first round of the Delphi survey, which was completed by 40 of the 42 panelists. The consensus for an agreement was achieved for 26 items, while 4 items failed to achieve any consensus. No item reached the consensus threshold for disagreement. There was one new item proposal that was added to the list after discussion by the steering committee (item#17, robustness assessment of end-to-end deep learning pipelines). A summary of the votes in Delphi Round#1 is presented in Fig. 3. The highest agreement (100%) was achieved by item#21 (i.e., consideration of uncertainty).

Following the Round#1, 4 items with no consensus and 1 newly proposed item were presented to the Group#2 panelists in Round#2 of the Delphi process. In this round, 41 of the 42 panelists participated. The consensus for an agreement was achieved for 4 items. There was no consensus on 1 item about prospective data collection, which was therefore removed from the list. There was no disagreement with consensus. A summary of the votes in Round#2 is presented in Fig. 3.

All Group#1 panelists were invited to Round#3 for the panel discussion by the steering committee members. A small number of minor modifications were made to the item definitions at this time. The agreement was achieved for all 30 items within 9 categories.

**Fig. 4:**
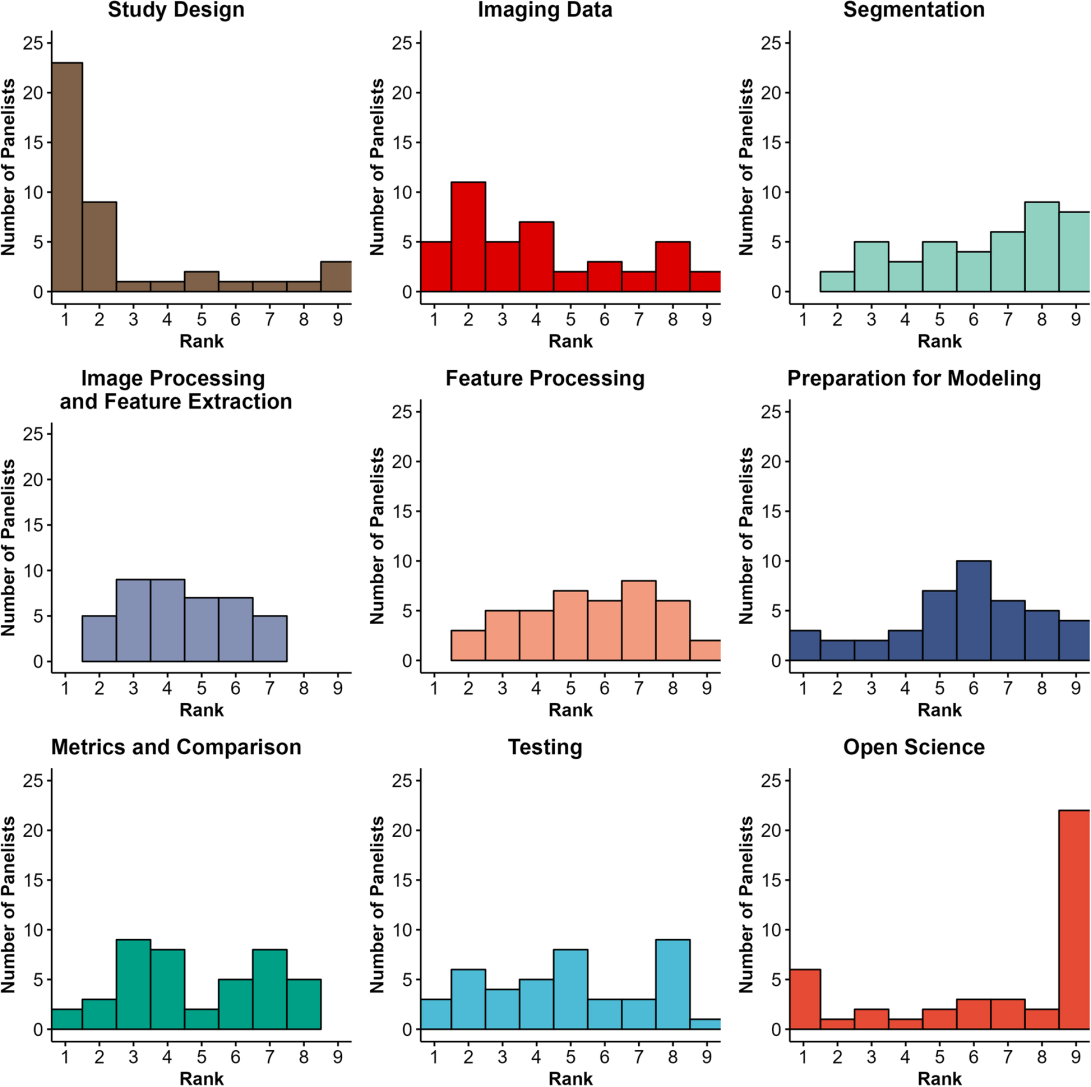
Histogram plots depicting total category rank counts as assigned by panelists. The closer a rank is to 1, the greater its relative importance

**Fig. 5:**
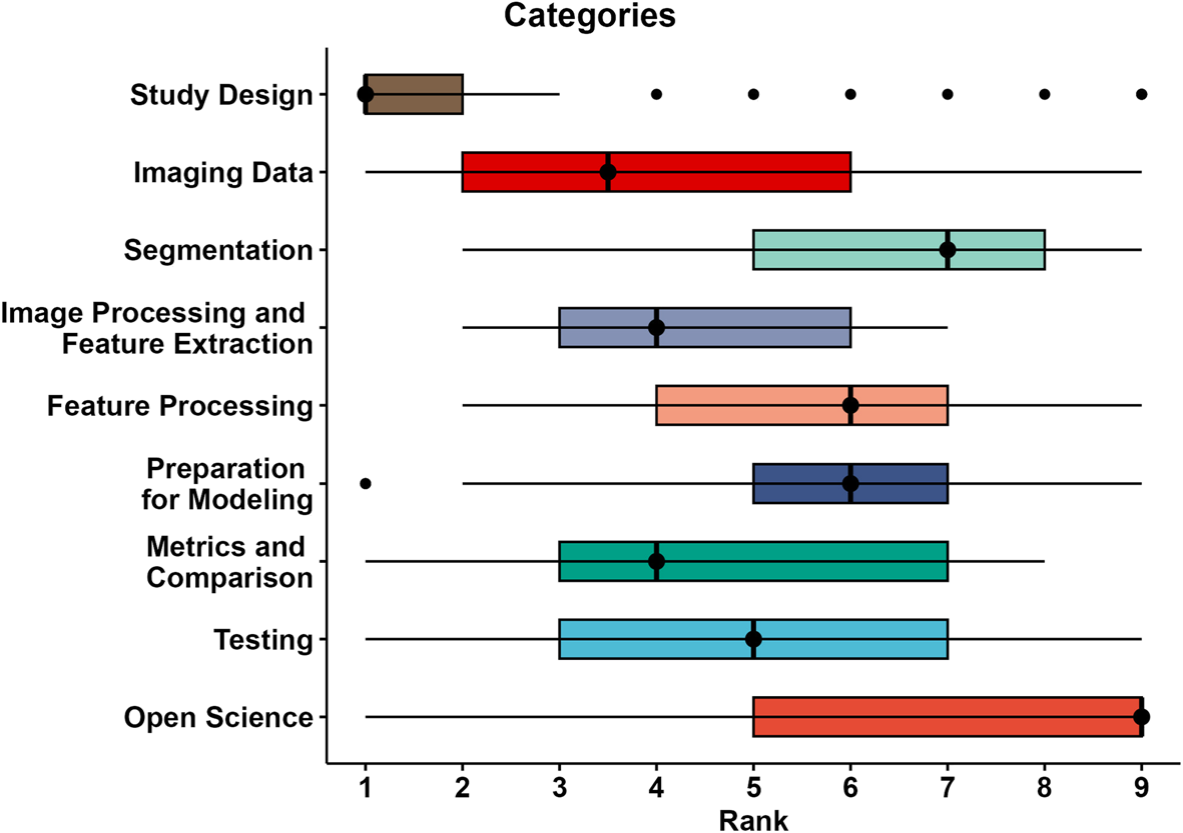
Box plots for rank statistics of categories. The closer a rank is to 1, the greater its importance. Shaded bars depict interquartile range

**Fig. 6:**
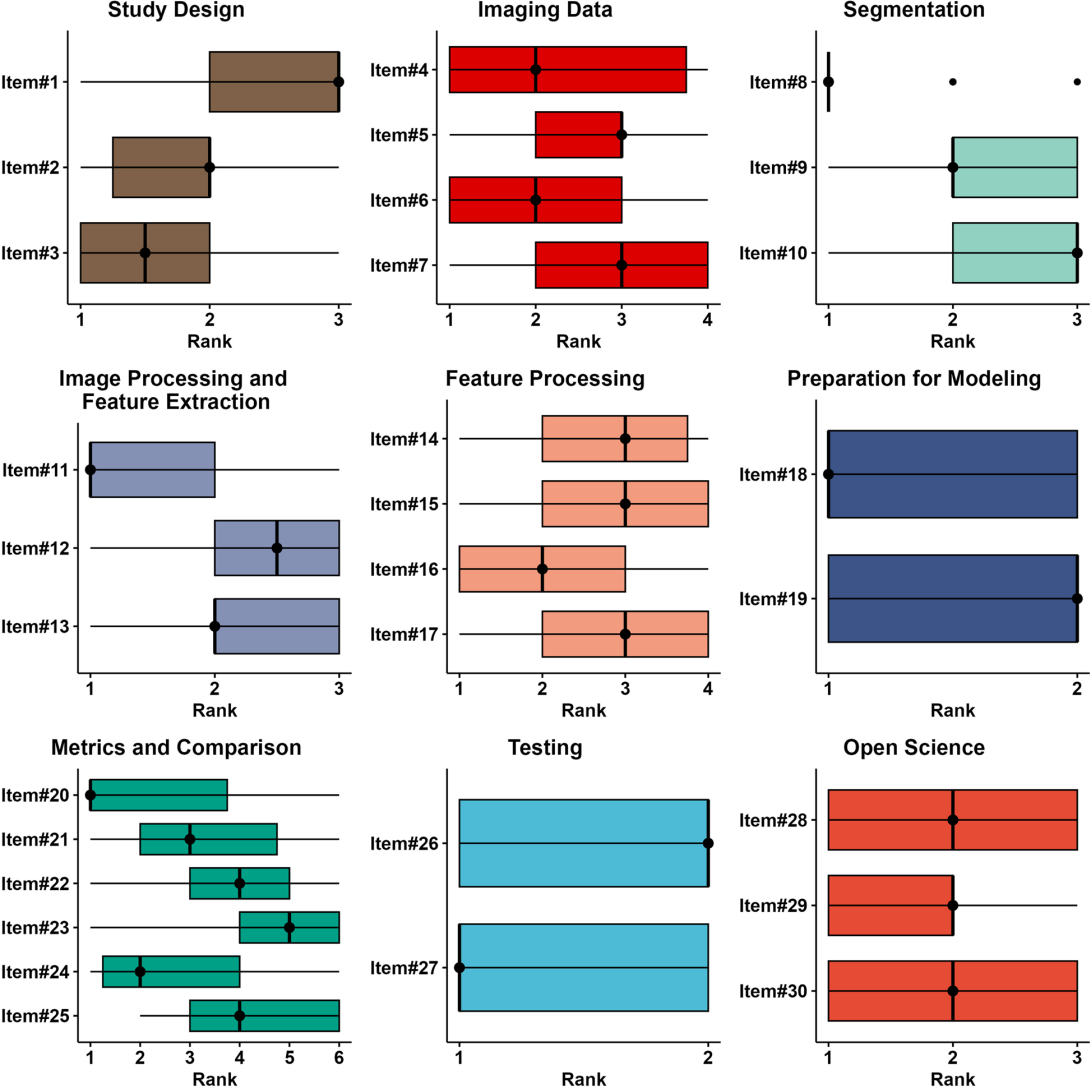
Box plots for rank statistics of items. The closer a rank is to 1, the greater its importance. Shaded bars depict interquartile range

The final Delphi round, Round#4, consisted of ranking of all 9 categories and the 30 items divided by category. This was performed by all 42 of the Group#2 panelists. Total category rank counts as assigned by panelists is presented in Fig. 4. A summary of the category and item ranks in Round#4 is presented in Figs 5 and 6, respectively.

**Fig. 7:**
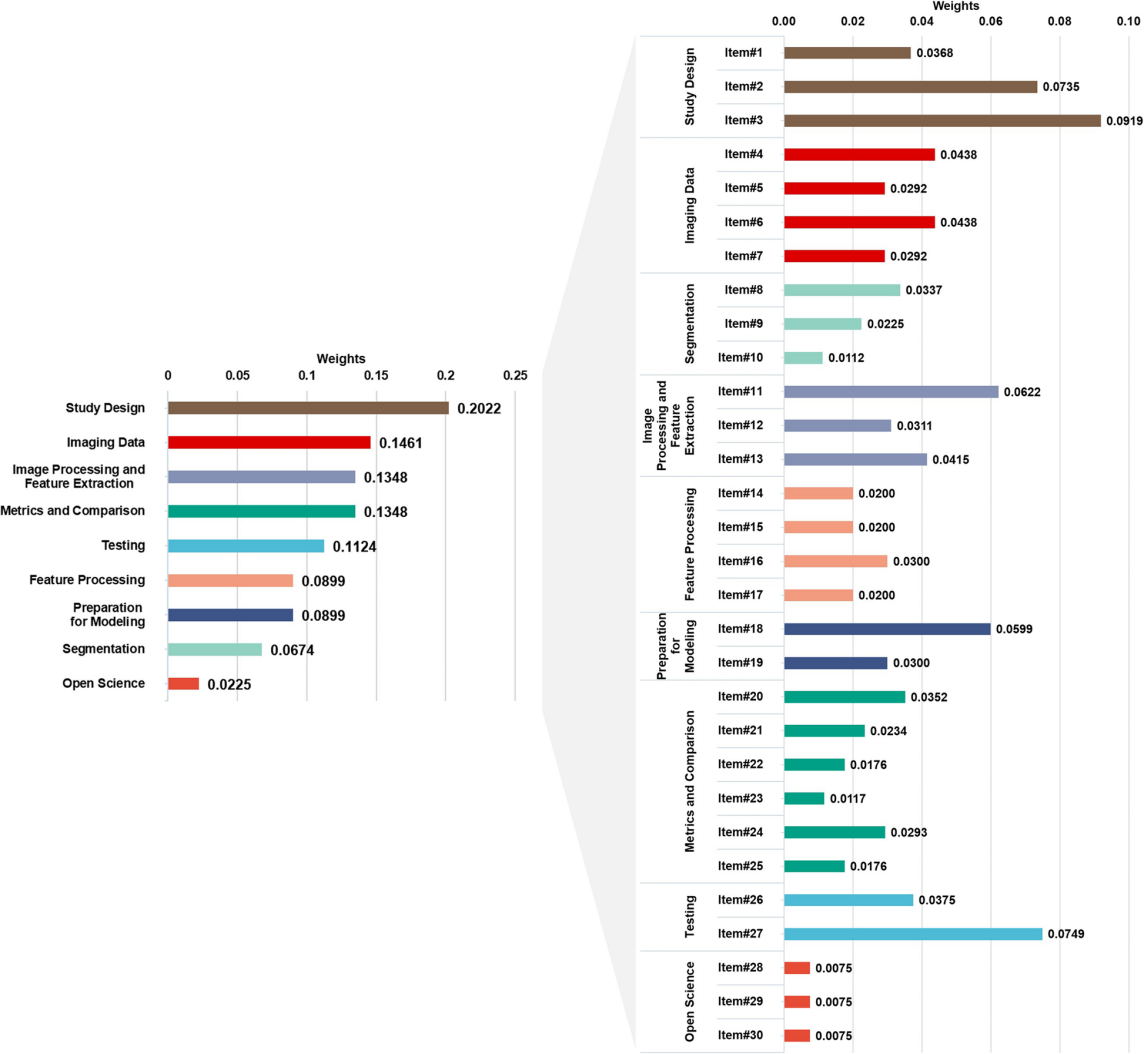
Weights of METRICS categories and items. Each category has a different color and those colors are matched between right and left panels

Weights calculated for categories and items are presented in Fig. 7. For categories, the highest and lowest weights belonged to study design and open science, respectively. According to their final weights, top 5 items with highest weights were as follows: item#3 (i.e., high-quality reference standard with a clear definition; weight, 0.0919); item#27 (i.e., external testing; weight, 0.0749); item#2 (i.e., eligibility criteria that describe a representative study population; weight, 0.0735); item#11 (i.e., appropriate use of image preprocessing techniques with transparent description; weight, 0.0622); and item#18 (i.e., proper data partitioning process; weight, 0.0599). The lowest weights belonged to the three items of category “open science” and were as follows: item#28 (i.e., data availability; weight, 0.0075), item#29 (i.e., code availability; weight, 0.0075), and item#30 (i.e., model availability; weight, 0.0075).

Anonymized individual votes and ranks obtained in the Round#1, Round#2, and Round#4 of the Stage#3 are presented in Supplementary file 1.

### Finalized METRICS tool

**Fig. 8:**
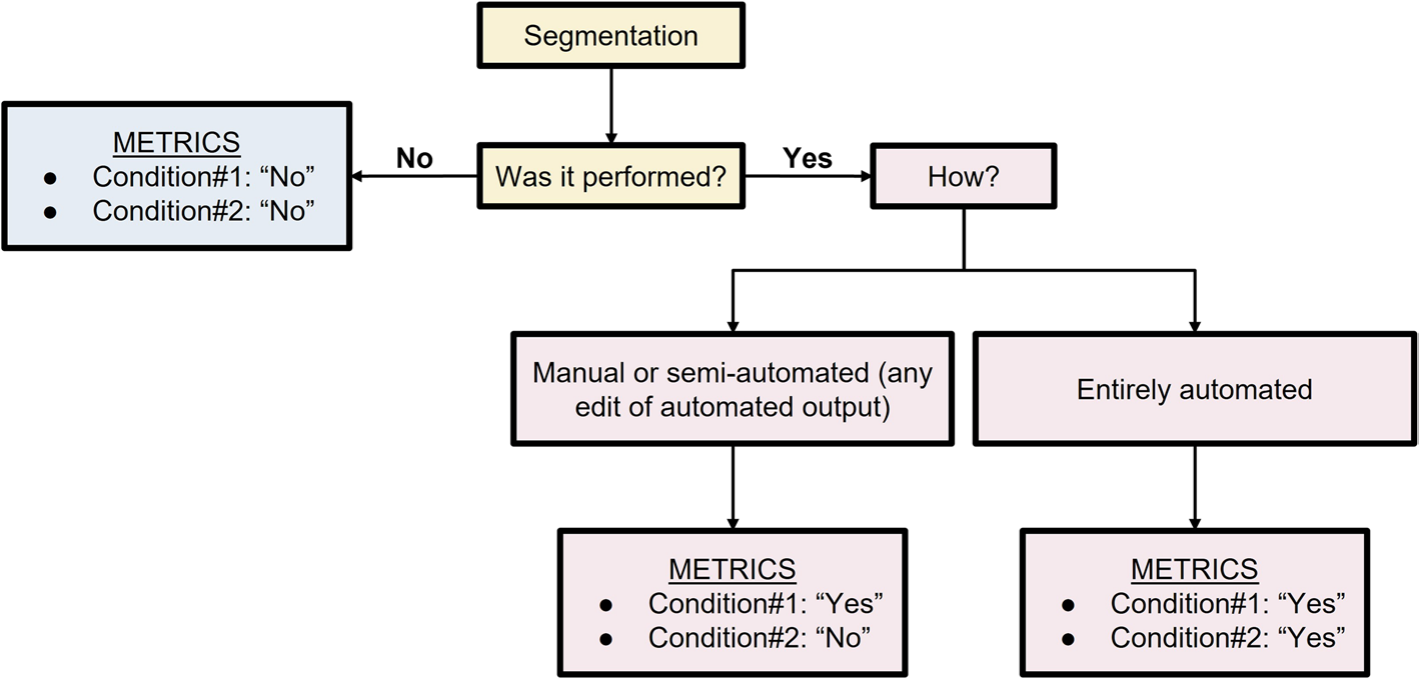
Use of conditions for the “Segmentation” section. Please note, the term “segmentation” refers to either fine (e.g., semantic, or pixel-based) or rough (e.g., cropping or bounding box) delineation of a region or volume of interest within an image or image stack for model training or evaluation. Studies can also be performed without such annotations, for example, using class labels that are assigned either to the entire image, volume, exam, or patient or with unsupervised approaches that require no labeling at all (e.g., clustering models)

**Fig. 9:**
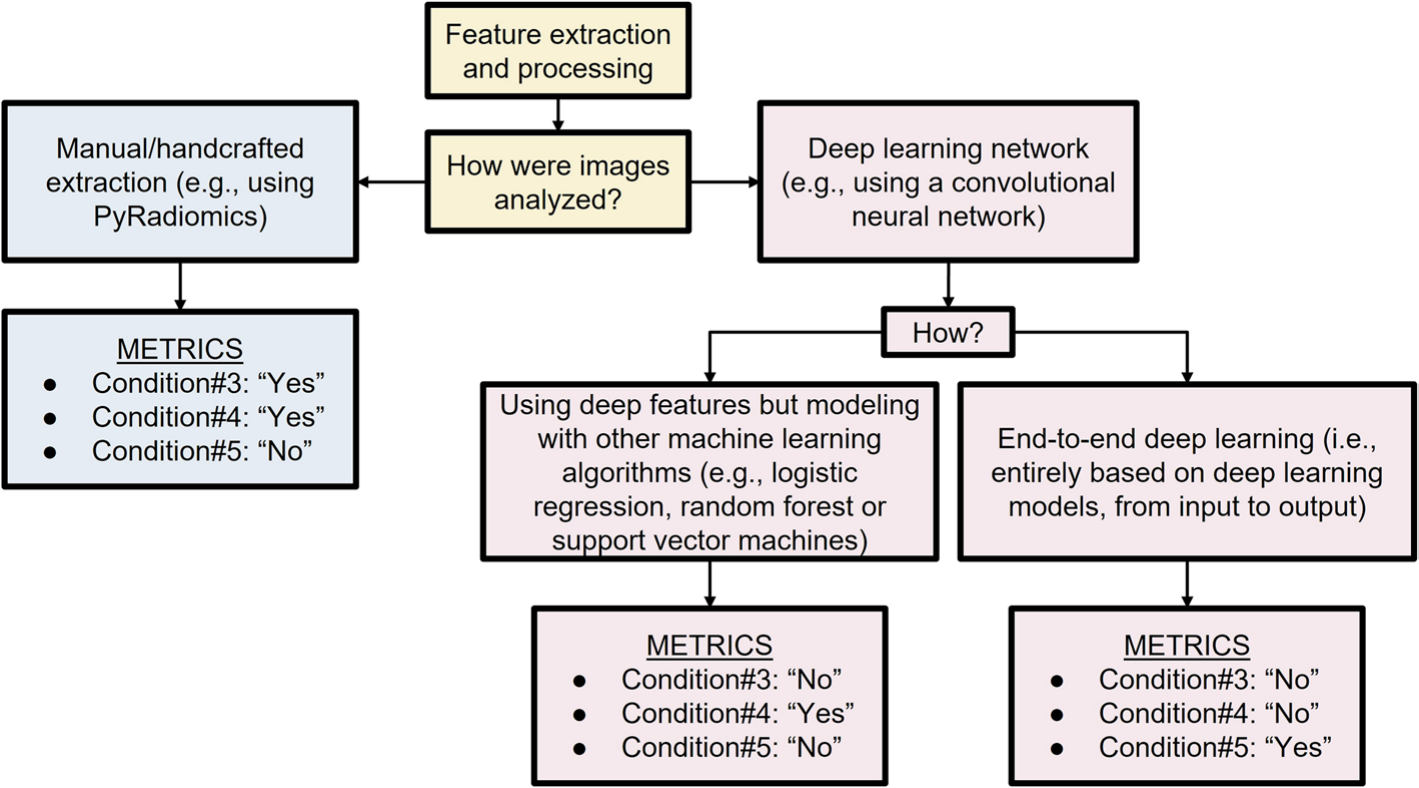
Use of conditions related to the sections “Image processing and feature extraction'' and “Feature processing”. Please note the flowchart assumes a single pipeline is used in a given study. However, different techniques might coexist in a single study. For instance, a study might include both hand-crafted feature extraction and end-to-end deep learning for comparison purposes, in such a case, all conditions can be selected as “Yes”

The final METRICS tool included 30 items within 9 categories and is presented in Table 1 with relative item weights. It also accounts for different study pipelines by including several conditional items. Figures 8 and 9 present a flow diagram to exemplify their usage in practice.

**Table 1:** METRICS tool

Categories	No.	Items	Weights	Score^f^
Study design	#1	Adherence to radiomics and/or machine learning-specific checklists or guidelines	0.0368	
	#2	Eligibility criteria that describe a representative study population	0.0735	
	#3	High-quality reference standard with a clear definition	0.0919	
Imaging data	#4	Multi-center	0.0438	
	#5	Clinical translatability of the imaging data source for radiomics analysis	0.0292	
	#6	Imaging protocol with acquisition parameters	0.0438	
	#7	The interval between imaging used and reference standard	0.0292	
Segmentation^a^	#8	Transparent description of segmentation methodology	0.0337	
	#9	Formal evaluation of fully automated segmentation^b^	0.0225	
	#10	Test set segmentation masks produced by a single reader or automated tool	0.0112	
Image processing and feature extraction	#11	Appropriate use of image preprocessing techniques with transparent description	0.0622	
	#12	Use of standardized feature extraction software^c^	0.0311	
	#13	Transparent reporting of feature extraction parameters, otherwise providing a default configuration statement	0.0415	
Feature processing	#14	Removal of non-robust features^d^	0.0200	
	#15	Removal of redundant features^d^	0.0200	
	#16	Appropriateness of dimensionality compared to data size^d^	0.0300	
	#17	Robustness assessment of end-to-end deep learning pipelines^e^	0.0200	
Preparation for modeling	#18	Proper data partitioning process	0.0599	
	#19	Handling of confounding factors	0.0300	
Metrics and comparison	#20	Use of appropriate performance evaluation metrics for task	0.0352	
	#21	Consideration of uncertainty	0.0234	
	#22	Calibration assessment	0.0176	
	#23	Use of uni-parametric imaging or proof of its inferiority	0.0117	
	#24	Comparison with a non-radiomic approach or proof of added clinical value	0.0293	
	#25	Comparison with simple or classical statistical models	0.0176	
Testing	#26	Internal testing	0.0375	
	#27	External testing	0.0749	
Open science	#28	Data availability	0.0075	
	#29	Code availability	0.0075	
	#30	Model availability	0.0075	
Total METRICS score (should be given as percentage)				
Quality category^g^				

^a^Conditional for studies including region/volume of interest labeling

^b^Conditional for studies using fully automated segmentation

^c^Conditional for the hand-crafted radiomics

^d^Conditional for tabular data use

^e^Conditional on the use of end-to-end deep learning

^f^Score is simply the weight if present and 0 otherwise

^g^Proposed total score categories: 0 ≤ score < 20%, “very low”; 20 ≤ score < 40%, “low”; 40 ≤ score < 60%, “moderate”; 60 ≤ score < 80%, “good”; and 80 ≤ score ≤ 100%, “excellent” quality

A user-friendly online calculation tool was prepared to streamline the calculation of the METRICS score (https://metricsscore.github.io/metrics/METRICS.html). It also allows printing (paper and PDF) and exporting (Excel spreadsheet). Supplementary file 2 (without explanation) and Supplementary file 3 (with explanation) allow downloading the METRICS tool in table format. However, the use of the online tool mentioned above is highly recommended, as the final METRICS percentage score is based on the maximum achievable absolute score after accounting for item conditionality. This calculation can be performed automatically by the web-based tools (both online and offline versions). Supplementary file 4 includes evaluation examples from the literature, covering the use of METRICS on different radiomics pipeline designs.

A GitHub repository was set up for the METRICS tool (https://github.com/metricsscore/metrics). The discussion function was activated to receive community feedback to improve it in the future. Also, an offline version of the calculation tool can be downloaded from this repository, which requires no setup or installation but directly starts working on common web browsers such as Google Chrome (recommended; Google LLC). The online calculation tool and potential updates can also be accessed via this repository.

### Total score categories

To improve the comprehensibility of the METRICS total score, we propose the use of 5 arbitrary categories as a representation of gradually increasing quality, namely, 0 ≤ score < 20%, “very low”; 20 ≤ score < 40%, “low”; 40 ≤ score < 60%, “moderate”; 60 ≤ score < 80%, “good”; and 80 ≤ score ≤ 100%, “excellent” quality. However, these categories should be validated through future systematic reviews using METRICS and used as a complement of the METRICS quantitative score and not as its substitute.

## Discussion

In this work, we developed a scoring tool for assessing the methodologic quality of the radiomics research, i.e., METRICS, based on the input of a diverse and large international panel with 59 participants. Our study was conducted in 3 consecutive stages, with 4 rounds of the modified Delphi exercise in the last stage. Based on panelist ratings, 30 items within 9 categories were ultimately included in the METRICS tool. The weights of these items were then calculated using a hierarchical ranking of categories and items based on the rank-based assessment by the Delphi panelists. A web application was developed to automate the calculation of the METRICS score, and a repository was created to collect feedback from the radiomics research community.

There have been only few tools proposed to assess the methodological quality of radiomics research in the literature, e.g., the RQS [10]. Despite the fact that the RQS was published as part of a review article, it has received so much attention from the community that it became the de facto standard for evaluating radiomics methodology [11]. Although it was developed and published by leading radiomics researchers, it lacked methodological transparency in terms of how it was developed and how the scores for each item were assigned. The first and most widely used version was designed to evaluate traditional radiomics and modeling in general and thus does not apply to deep learning workflows. Although not directly related to radiomics, the Must AI Criteria-10 (MAIC-10) checklist can be used to evaluate the quality of artificial intelligence (AI) and medical imaging studies [36]. It aims to simplify the process while overcoming some of the limitations of other published checklists in the fields of artificial intelligence and medical imaging. MAIC-10 is a very short and simple tool that covers a wide range of concepts. According to the authors of MAIC-10, unlike other checklists or quality scoring tools, it was designed to provide a quantitative, objective, and reproducible quality score with a broad scope of applications across studies on AI in medical imaging. MAIC-10 achieved a high correlation score to Checklist for Artificial Intelligence in Medical Imaging (CLAIM) [27], a widely used 42-item reporting checklist, despite being tested on a small number of publications from the journal in which it was published. It was also proposed as the most reproducible checklist in terms of intra-observer reproducibility, with CLAIM taking second place. However, the MAIC-10 scores are unweighted, namely ignoring the relative importance of each item and simply assigning a score of 1 for adherence. Such a simple scoring strategy was also used for the Transparent Reporting of a multivariable prediction model for Individual Prognosis Or Diagnosis (TRIPOD) checklist as well [37]. A recent radiomics-specific reporting checklist, the CLEAR checklist, was developed by an international initiative led by a group of experts and endorsed by ESR and EuSoMII [15]. Although CLEAR was designed primarily as a reporting tool and not a methodological guide, it still provides useful information about the methodology. Furthermore, it has a shortened version called CLEAR-S that focuses solely on methodological aspects and open science, with no score or weights. There are also reporting checklists for AI and medical imaging that were not specifically designed for radiomics, such as CLAIM [27]. CLAIM is a highly cited checklist that provides guidance for reporting and methodology. However, the current version of the CLAIM was created by a relatively small group of scientists with no formal methodology for determining item eligibility, such as the Delphi method; nevertheless, there is a further initiative ongoing to update CLAIM [38]. Of note, a recent article provides a comprehensive review of available guidelines that can be used in AI research and medical imaging [39].

To develop the proposed scoring system, we used a modified Delphi method with an international group of panelists and defined weights of each item to present a more nuanced way of assessment. As a result, the category “Study design” had the highest weight and thus the biggest effect on the final score. This result is such that adhering to all items of the category may already allow a METRICS score ranging between 20% and 25%, considering all possible conditionals. It includes three items as follows: *i*, adherence to radiomics and/or machine learning-specific checklists or guidelines; *ii*, eligibility criteria that describe a representative study population; and *iii*, high-quality reference standard with a clear definition. The first item was introduced as a new concept in comparison to the RQS [10] and MAIC-10 [36] tools. The authors of the MAIC-10 checklist included the study design as a single item and defined it as a very broad concept. While most of their 10 items were discussed in at least half of the studies evaluated as part of the MAIC-10, the study design was not defined in any of the studies evaluated. Previously, the CLEAR checklist [15] and, to a lesser extent, CLAIM [27] drew attention to some of these concepts in terms of reporting.

It may appear surprising that the category related to open science practices had the lowest weight and thus the lowest effect on the final score. This result, however, should be intended to only reflect relative weights between METRICS categories and by no means as a general disregard for open science. The very presence of these items in METRICS, after all, attests that panelists reached a consensus on the necessity of their inclusion. As widely known, radiomic studies suffer from significant reproducibility and replicability issues, which have been mainly attributed to the lack of data, code, and model sharing practices leading to poor generalizability [8, 40–42]. The METRICS authors strongly believe that open science practices should be followed in order to address these limitations and facilitate radiomics implementation into clinical practice. The discrepancy between these considerations and the assigned weights may be attributable to the assessment that proper study design “comes first”. In other words, if the study’s aims and methodological steps are flawed, data and model availability becomes a secondary concern as these studies would still lack value in the clinical setting. It should also be noted that reproducibility, replicability, and generalizability are complex, intertwined topics and not exclusive to the field of radiomics, and reliable solutions to satisfactorily address them are still being investigated [43]. We consider that scoring highly on METRICS will not only mean an experiment has been correctly designed and presented but that these same aspects will also ultimately improve its reproducibility, replicability, and generalizability.

Even though an item focused on the role of prospective study design/data collection was initially included, the panelists were unable to reach an agreement on it, and it is not present in the final METRICS tool. The RQS, on the other hand, places a strong emphasis on prospective studies, particularly those registered in trial databases, and awards the studies with the highest score of the tool for this item [10]. Based on the feedback of the panelists during Round#1 of Stage#3, the most likely reason for this would be that radiomics research requires large data sets, which are difficult to achieve with prospective studies when compared to retrospective design and data sets. Another issue raised by panelists was the potential penalization of large retrospective data sets in comparison to prospective studies with small data sets. Therefore, despite its undoubtedly high importance in clinical research, the role and added value of prospective data collection currently remain uncertain in radiomics and artificial intelligence research within the medical imaging domain and could be secondary compared to other considerations on overall data labeling and management as established by the METRICS expert panel. It would be worthwhile to receive community feedback on this and other topics in the future, which may contribute to future revisions of METRICS.

It should be noted that METRICS not only has the potential to improve the quality of the radiomic research papers but also to serve as a development model for future standardization and evaluation tools. Nevertheless, to facilitate clinical translation of radiomics, further endeavors and standardization projects are still required. Examples of previous standardization attempts include the IBSI [28] and the CLEAR checklist [15]. These efforts should also include altering the attitudes of academic journals towards negative results, i.e., statistically non-significant results [3, 6], promoting the use of checklists and quality scoring tools [44], and fostering the conduct of reproducibility studies. Particular attention should also be posed to trending research practices which may not only be unrealistic for the clinical setting but also methodologically inappropriate, such as the use of radiomics-based nomograms [45]. Finally, METRICS is explicitly targeted at the research setting, while commercially available products based on radiomics and machine learning have to account for further issues such as regulatory demands and liability for potential mistakes, which are outside the scope of the tool we developed.

Our work has several distinguishing features and strengths compared to the previously available tool. First, some RQS items, such as requirement for phantom-based test-retest experiments or scanning at multiple time points should not be expected in all radiomics studies. Second, we assigned weights for items and categories based on expert ranking and not arbitrarily. This was one of the main goals of the study as there has been no previous work on radiomics quality scoring that has presented a transparent methodology for assigning item weights. Third, the METRICS tool considers not only hand-crafted radiomics but also deep learning-based radiomics. Fourth, both Group#1 and Group#2 had a large number of panelists. Furthermore, the panel was diverse in terms of country and domain expertise. This was necessary to reduce noise in calculations. Fifth, panelist participation in the Delphi rounds was also very high, with a minimum of 95% (40 of 42). Sixth, we created an easy-to-use web application to streamline scoring. This was crucial because METRICS contains conditional items that cover all aspects of radiomics, which may make the calculation difficult on paper. Finally, we established a living repository to discuss the METRICS tool and its content and receive feedback in order to improve them in the future.

There are however several limitations to declare. First, our modified Delphi procedure was not completely anonymous and the steering committee had access to identities, which was a deviation from the standard Delphi exercise. We chose this approach to ensure panelist participation. Nevertheless, we kept the votes and comments anonymous for other panelists. Second, a systematic or quantitative strategy, such as considering publication metrics, was not employed in the selection of the panelists (particularly, the Group#2 panelists). Our efforts were focused on assembling a diverse group of knowledgeable figures in the fields of radiomics and informatics, including editors and members of editorial boards from publications that commonly publish works relating to these topics. To represent different stakeholders in medical imaging, the panelists also included prominent figures having strong backgrounds in both radiology and nuclear medicine, as well as non-physicians. Geographical location of the panelists was not a factor in their participation; as a result, the representation of different countries within the author group presents some degree of imbalance. Third, the ranking in Round#4 of Stage#3 did not account for potential items of equal importance. An analytical hierarchy process and pairwise voting could have been an alternative approach that takes equality into account. However, by this method, the number of questions would have been doubled in Round#4, which might have caused fatigue and had negative effects on the scoring process. Fourth, during tool development, the need for conditional items became apparent, even if their use may complicate the scoring process. In reality, radiomic research involves numerous methodological variations and nuances that could be overlooked with a fixed item list. However, the availability of online and offline automated calculation tools should help mitigate this limitation. Fifth, the conditionality of the items or categories was not taken into account when calculating weights. Dynamic weights would have necessitated calculations of all possible conditional combinations and, as a result, multiple rankings, which is impractical and of limited value as differences are expected to be small compared to the current METRICS tool. Sixth, the number of items in each category varied. Nonetheless, the weighting process accounted for this to avoid biases in the final tool due to item number within categories. Seventh, the order of the items and categories in the Delphi rounds was fixed, which may have an influence on ranking and introduce bias. Alternatively, the order of these could have been randomized during voting, and this could have been done independently for each panelist as well. Finally, the reproducibility of the METRICS was not evaluated. Such an analysis necessitates a dedicated study design by incorporation of other tools for comparison, which should be performed in a future investigation.

In conclusion, we developed a scoring tool for a comprehensive assessment of the methodologic quality of the radiomics research, i.e., METRICS, with a large international panel of experts and by using a modified Delphi protocol. With its flexible format to cover all methodological variations, it provides a well-constructed framework for the key methodological concepts to assess the quality of the radiomic research papers. A web application was developed to help with the calculation of the METRICS score, and a repository was created to collect feedback from the radiomics community. We hope that the researchers would benefit from this tool when designing their studies, assessing the methodological quality of papers in systematic reviews, and that journals would adopt the METRICS quality scoring tool for peer review. Comments and contributions to this tool are welcome through its repository to improve it in the future.

### Supplementary Information

**Additional file 1.** 

**Additional file 2.** 

**Additional file 3.** 

**Additional file 4.** 

## Data Availability

Data generated or analyzed during this study are presented with this manuscript.

## Abbreviations

AI: Artificial intelligence
CLAIM: Checklist for Artificial Intelligence in Medical Imaging
CLEAR: CheckList for EvaluAtion of Radiomics research
ESR: European Society of Radiology
EuSoMII: European Society of Medical Imaging Informatics
MAIC-10: Must AI Criteria-10 checklist
METRICS: METhodological RadiomICs Score
RQS: Radiomics quality score
TRIPOD: Transparent reporting of a multivariable prediction model for individual prognosis or diagnosis
