# Supplementary material for: METhodological RadiomICs Score (METRICS): a quality scoring tool for radiomics research endorsed by EuSoMII
Source: Insights Imaging. 2024 Jan 17;15:8. doi: 10.1186/s13244-023-01572-w (PMC10792137; doi:10.1186/s13244-023-01572-w)
Supplement: Supplementary file 2 — Additional file 2. [file 13244_2023_1572_MOESM2_ESM.docx]

**METhodological RadiomICs Score (METRICS): A quality scoring tool for radiomics research endorsed by EuSoMII**

**ELECTRONIC SUPPLEMENTARY MATERIAL**

**Supplementary file 2:** METRICS tool without explanations.

| **Categories** | **No.** | **Items** | **Weights** | **Score**^6^ |
| --- | --- | --- | --- | --- |
| Study Design | #1 | Adherence to radiomics and/or machine learning-specific checklists or guidelines | 0.0368 |  |
|  | #2 | Eligibility criteria that describe a representative study population | 0.0735 |  |
|  | #3 | High-quality reference standard with a clear definition | 0.0919 |  |
| Imaging Data | #4 | Multi-center | 0.0438 |  |
|  | #5 | Clinical translatability of the imaging data source for radiomics analysis | 0.0292 |  |
|  | #6 | Imaging protocol with acquisition parameters | 0.0438 |  |
|  | #7 | The interval between imaging used and reference standard | 0.0292 |  |
| Segmentation^1^ | #8 | Transparent description of segmentation methodology | 0.0337 |  |
|  | #9 | Formal evaluation of fully automated segmentation^2^ | 0.0225 |  |
|  | #10 | Test set segmentation masks produced by a single reader or automated tool | 0.0112 |  |
| Image Processing and Feature Extraction | #11 | Appropriate use of image preprocessing techniques with transparent description | 0.0622 |  |
|  | #12 | Use of standardized feature extraction software^3^ | 0.0311 |  |
|  | #13 | Transparent reporting of feature extraction parameters, otherwise providing a default configuration statement | 0.0415 |  |
| Feature Processing | #14 | Removal of non-robust features^4^ | 0.0200 |  |
|  | #15 | Removal of redundant features^4^ | 0.0200 |  |
|  | #16 | Appropriateness of dimensionality compared to data size^4^ | 0.0300 |  |
|  | #17 | Robustness assessment of end-to-end deep learning pipelines^5^ | 0.0200 |  |
| Preparation for Modeling | #18 | Proper data partitioning process | 0.0599 |  |
|  | #19 | Handling of confounding factors | 0.0300 |  |
| Metrics and Comparison | #20 | Use of appropriate performance evaluation metrics for task | 0.0352 |  |
|  | #21 | Consideration of uncertainty | 0.0234 |  |
|  | #22 | Calibration assessment | 0.0176 |  |
|  | #23 | Use of uni-parametric imaging or proof of its inferiority | 0.0117 |  |
|  | #24 | Comparison with a non-radiomic approach or proof of added clinical value | 0.0293 |  |
|  | #25 | Comparison with simple or classical statistical models | 0.0176 |  |
| Testing | #26 | Internal testing | 0.0375 |  |
|  | #27 | External testing | 0.0749 |  |
| Open Science | #28 | Data availability | 0.0075 |  |
|  | #29 | Code availability | 0.0075 |  |
|  | #30 | Model availability | 0.0075 |  |
| Total METRICS score (should be given as percentage) | | | |  |
| Quality category^7^ | | | |  |

^1^ Conditional for studies including region/volume of interest labeling. ^2^ Conditional for studies using fully automated segmentation. ^3^ Conditional for the hand-crafted radiomics. ^4^ Conditional for tabular data use. ^5^ Conditional on the use of end-to-end deep learning.^6^ Score is simply the weight if present and 0 otherwise. ^7^ Proposed total score categories: 0≤score<20%, “very low”; 20≤score<40%, “low”; 40≤score<60%, “moderate”; 60≤score<80%, “good”; and 80≤score≤100%, “excellent” quality.
