# Supplementary material for: METhodological RadiomICs Score (METRICS): a quality scoring tool for radiomics research endorsed by EuSoMII
Source: Insights Imaging. 2024 Jan 17;15:8. doi: 10.1186/s13244-023-01572-w (PMC10792137; doi:10.1186/s13244-023-01572-w)
Supplement: Supplementary file 4 — Additional file 4. [file 13244_2023_1572_MOESM4_ESM.docx]

**METhodological RadiomICs Score (METRICS): A quality scoring tool for radiomics research endorsed by EuSoMII**

**ELECTRONIC SUPPLEMENTARY MATERIAL**

**Supplementary file 4:** Evaluation examples for the demonstration of how to use METRICS. The table reports whether each of the example studies fulfill (“yes”) or not (“no”) the item requirements or if the item is not applicable to the study design (“n/a”), as appropriate. Please note that conditional item weights do not influence the maximum obtainable total score in case of non-applicability.

| **Items/Conditions** | **Weights** | **Cuocolo (2021)^1^** | **Gitto (2021)^2^** | **Kobayashi (2021)^3^** |
| --- | --- | --- | --- | --- |
| Study Design |  |  |  |  |
| Item#1 | 0.0368 | no | no | no |
| Item#2 | 0.0735 | yes | yes | yes |
| Item#3 | 0.0919 | yes | yes | yes |
| Imaging Data |  |  |  |  |
| Item#4 | 0.0438 | no | yes | yes |
| Item#5 | 0.0292 | no | yes | yes |
| Item#6 | 0.0438 | yes | yes | no |
| Item#7 | 0.0292 | yes | yes | no |
| Segmentation |  |  |  |  |
| Condition#1 |  | yes | yes | yes |
| Condition#2 |  | yes | no | yes |
| Item#8 | 0.0337 | yes | yes | yes |
| Item#9 | 0.0225 | yes | n/a | yes |
| Item#10 | 0.0112 | yes | yes | yes |
| Image Processing and Feature Extraction |  |  |  |  |
| Condition#3 |  | no | yes | no |
| Item#11 | 0.0622 | yes | yes | yes |
| Item#12 | 0.0311 | n/a | yes | n/a |
| Item#13 | 0.0415 | no | yes | yes |
| Feature Processing |  |  |  |  |
| Condition#4 |  | no | yes | yes |
| Condition#5 |  | yes | no | no |
| Item#14 | 0.0200 | n/a | yes | no |
| Item#15 | 0.0200 | n/a | yes | no |
| Item#16 | 0.0300 | n/a | no | no |
| Item#17 | 0.0200 | no | n/a | n/a |
| Preparation for Modeling |  |  |  |  |
| Item#18 | 0.0599 | yes | yes | yes |
| Item#19 | 0.0300 | no | no | no |
| Metrics and Comparison |  |  |  |  |
| Item#20 | 0.0352 | yes | yes | yes |
| Item#21 | 0.0234 | yes | no | yes |
| Item#22 | 0.0176 | no | yes | no |
| Item#23 | 0.0117 | yes | yes | no |
| Item#24 | 0.0293 | yes | yes | no |
| Item#25 | 0.0176 | no | no | no |
| Testing |  |  |  |  |
| Item#26 | 0.0375 | yes | no | no |
| Item#27 | 0.0749 | no | yes | no |
| Open Science |  |  |  |  |
| Item#28 | 0.0075 | yes | no | yes |
| Item#29 | 0.0075 | no | no | yes |
| Item#30 | 0.0075 | no | yes | no |
| METRICS score | | 63.7% | 80.1% | 57.2% |
| METRICS score category | | Good | Excellent | Moderate |

^1^Cuocolo R, Comelli A, Stefano A, Benfante V, Dahiya N, Stanzione A, Castaldo A, De Lucia DR, Yezzi A, Imbriaco M. Deep Learning Whole-Gland and Zonal Prostate Segmentation on a Public MRI Dataset. J Magn Reson Imaging. 2021 Aug;54(2):452-459. doi: 10.1002/jmri.27585. Epub 2021 Feb 26. PMID: 33634932.

^2^Gitto S, Cuocolo R, van Langevelde K, van de Sande MAJ, Parafioriti A, Luzzati A, Imbriaco M, Sconfienza LM, Bloem JL. MRI radiomics-based machine learning classification of atypical cartilaginous tumour and grade II chondrosarcoma of long bones. EBioMedicine. 2022 Jan;75:103757. doi: 10.1016/j.ebiom.2021.103757. Epub 2021 Dec 18. PMID: 34933178; PMCID: PMC8688587.

^3^Kobayashi K, Miyake M, Takahashi M, Hamamoto R. Observing deep radiomics for the classification of glioma grades. Sci Rep. 2021 May 25;11(1):10942. doi: 10.1038/s41598-021-90555-2. PMID: 34035410; PMCID: PMC8149679.
